# Tonsillectomy Improved Therapeutic Response in Anti-SRP Myopathy With Chronic Tonsillitis

**DOI:** 10.3389/fimmu.2020.595480

**Published:** 2020-11-24

**Authors:** Takuya Ikeda, Hideyuki Takeuchi, Keita Takahashi, Haruko Nakamura, Misako Kunii, Atsuko Katsumoto, Mikiko Tada, Yuichi Higashiyama, Takashi Hibiya, Shigeaki Suzuki, Ichizo Nishino, Shigeru Koyano, Hiroshi Doi, Fumiaki Tanaka

**Affiliations:** ^1^ Department of Neurology and Stroke Medicine, Yokohama City University Graduate School of Medicine, Yokohama, Japan; ^2^ Department of Pathology, Yokohama City University Hospital, Yokohama, Japan; ^3^ Department of Neurology, Keio University School of Medicine, Tokyo, Japan; ^4^ Department of Neuromuscular Research, National Institute of Neuroscience, and Department of Genome Medicine Development, Medical Genome Center, National Center of Neurology and Psychiatry, Tokyo, Japan

**Keywords:** anti-SRP myopathy, chronic tonsillitis, IgA nephritis, intravenous immunoglobulin, tonsillectomy

## Abstract

Chronic tonsillitis has been attracted attention as a source of abnormal immune responses and a possible trigger of autoimmune diseases such as IgA nephritis, IgA vasculitis, palmoplantar pustulosis, psoriasis, rheumatoid arthritis, Behçet’s disease, and myositis. Here we present the first report of anti–signal recognition particle antibody–associated necrotizing myopathy (anti-SRP myopathy) with IgA nephropathy and chronic tonsillitis in which the therapeutic response to intravenous immunoglobulin (IVIG) treatment was dramatically improved after tonsillectomy and accompanied by a rapid increase in ΔIgG, defined as the change in serum IgG levels 2 weeks after the start of IVIG treatment relative to pre-treatment levels. Moreover, serum anti-SRP antibody titers became undetectable after tonsillectomy even though the resected tonsils did not produce anti-SRP antibodies. Tonsillectomy should be considered when chronic tonsillitis is observed in patients with autoimmune diseases showing poor response to treatment, including anti-SRP myopathy.

## Introduction

Anti-signal recognition particle antibodies–associated necrotizing myopathy (anti-SRP myopathy) accounts for approximately one-third of immune-mediated necrotizing myopathies (IMNMs). It is typically characterized by rapidly progressive proximal muscle weakness and dysphagia with markedly elevated levels of creatine kinase (CK) and poor response to conventional immunosuppressive therapies. (1, 2)

IgA nephropathy is often accompanied by chronic tonsillitis as a source of abnormal immune reactions. (3) Tonsillectomy has been used to ablate disease activity although its therapeutic efficacy remains controversial. (4, 5) Here we report a case of anti-SRP myopathy with IgA nephropathy and chronic tonsillitis in which therapeutic response was dramatically improved with tonsillectomy.

## Case Report

**Figure 1:**
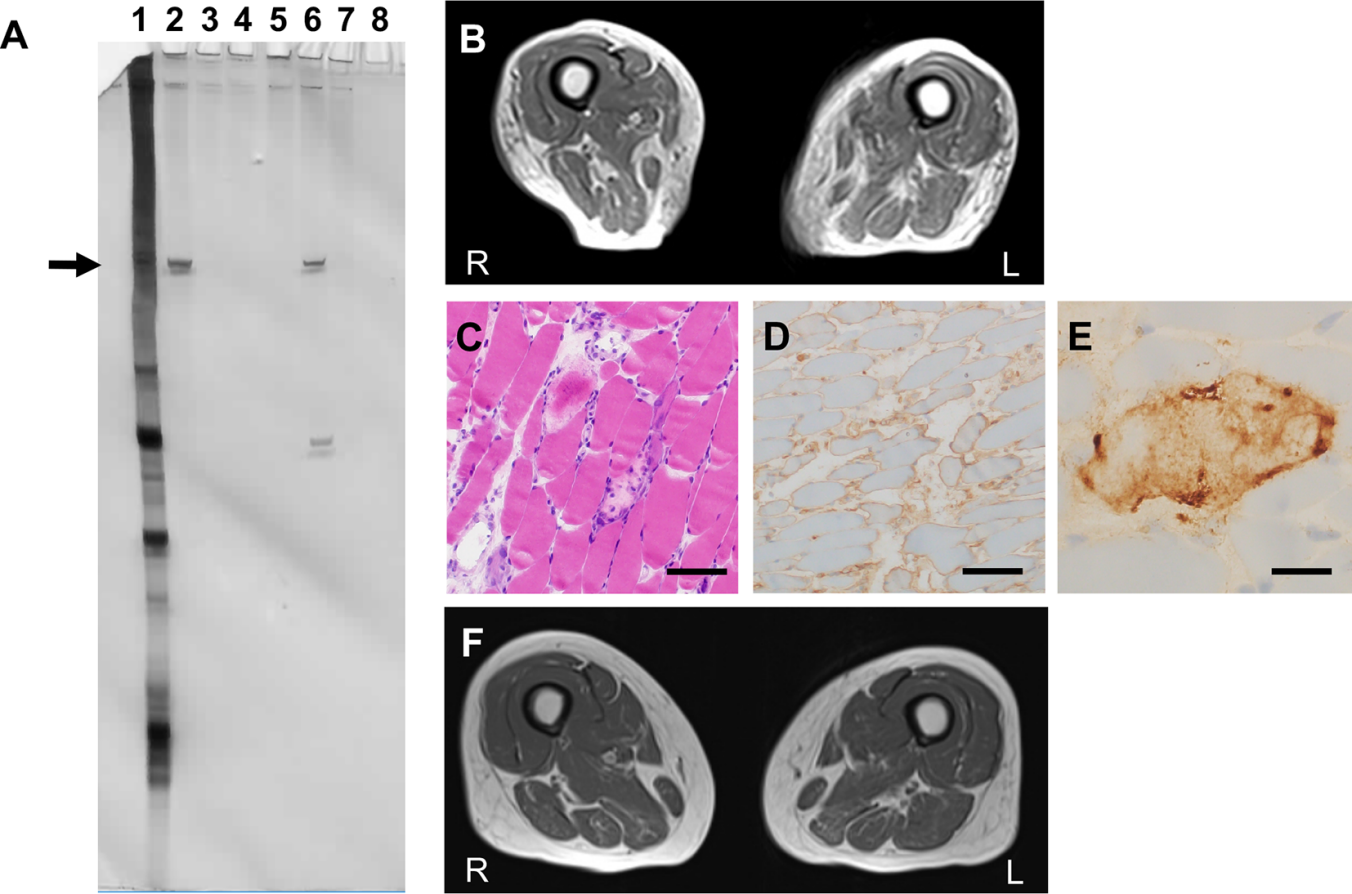
Clinical features **(A)** RNA immunoprecipitation assay to detect anti-signal recognition particle (SRP) antibodies. Serum and tonsil tissue from a patient with chronic tonsillitis was used as control. Each tonsil was ultrasonicated and lysed in radioimmunoprecipitation assay (RIPA) buffer [50 mM Tris-HCl at pH 7.5, 150 mM NaCl, 5 mM ethylenediaminetetraacetic acid (EDTA), 0.5% sodium deoxycholate, 1% NP-40, and 0.1% sodium dodecyl sulfate (SDS)] and a protease inhibitor cocktail (Complete mini EDTA-free, Roche) at a concentration of 40 μg/ml. Anti-SRP antibodies were detected using an RNA immunoprecipitation assay with extracts of HeLa cells as previously described. Briefly, 10 μl of serum or tonsil lysate was mixed with 2 mg of protein A-Sepharose CL-4B (Pharmacia Biotech AB) in 500 μl of immunoprecipitation buffer and incubated for 2 h. After washing three times with immunoprecipitation buffer, antigen-bound Sepharose beads were mixed with 100 μl of HeLa cell extract (6 × 10 cells equivalent per sample) for 2 h. Next, 30 μl of 3 M sodium acetate, 30 μl of 10% SDS, and 300 μl of phenol:chloroform:isoamyl alcohol (50:50:1, containing 0.1% 8-hydroxyquinoline) were added to extract the bound RNA. After ethanol precipitation, the RNA was resolved using a 7 M urea-8% polyacrylamide gel. The gel was silver-stained (Bio-Rad). Immunoprecipitated RNA located in the 7S RNA lesion (arrow) was regarded as anti-SRP antibody. Lane 1, total RNA; lane 2, patient’s serum; lane 3, patient’s tonsil lysate; lane 4, control serum; lane 5, control tonsil lysate; lane 6, positive control; lane 7, negative control; lane 8, empty. **(B)** Axial T1-weighted femoral MRI on admission showing severe femoral muscle atrophy with fat replacement. **(C–E)** Muscle biopsy sections of the left biceps. **(C)** Hematoxylin and eosin staining showed necrotizing myopathy with active necrosis and regeneration of muscle fibers with no lymphocyte infiltration. Scale bar, 100 μm. **(D)** Human leukocyte antigen–ABC staining indicated positive immunoreactivity in non-necrotic muscle fibers. Scale bar, 100 μm. **(E)** C5b-9 membrane attack complex staining exhibited immunopositive deposition on the surface of muscle fibers. Scale bar, 20 μm. **(F)** Axial T1-weighted femoral MRI on discharge showing recovery of muscular volume compared to **(B)**.

**Figure 2:**
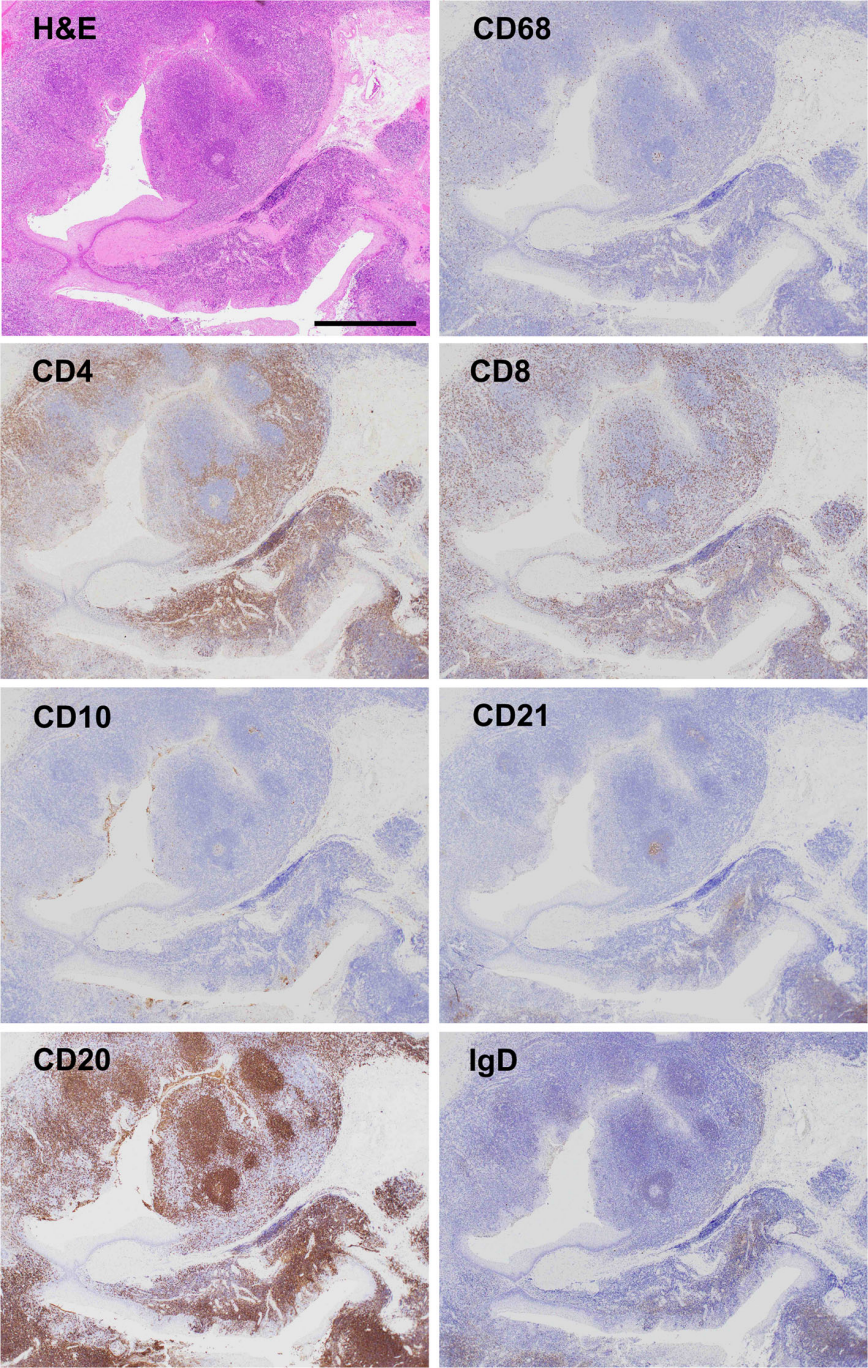
Micrographs of resected tonsil sections Hematoxylin and eosin (H&E) staining showed chronic tonsillitis with activated macrophages based on CD68 positivity. Immunostaining for CD4 and CD8 displayed T cell accumulation in the opposite sides of blind crypts. Most lymphoid follicles (CD20, a marker for follicular B cell) consisted of primary follicles with poorly developed germinal centers (CD10, a marker for germinal center B cells; CD21, a marker for germinal center dendritic cells), rare secondary follicle formation, and an expanded mantle zone (IgD, a marker for mantle zone B cells), which are characteristic pathological features of chronic tonsillitis associated with IgA nephritis but not chronic tonsillitis without IgA nephritis. Scale bar, 1 mm.

**Figure 3:**
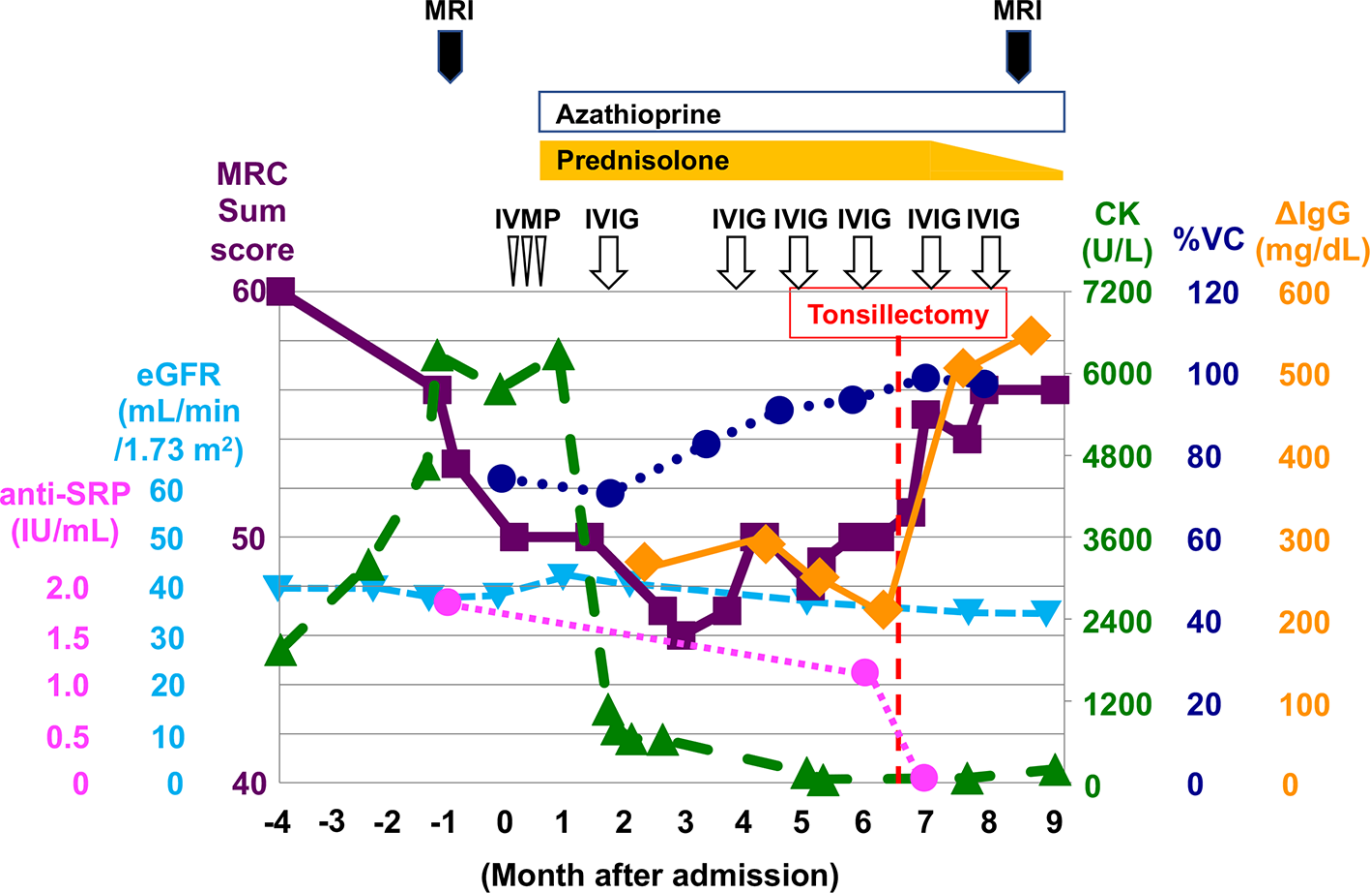
Clinical course and treatments during hospitalization. Tonsillectomy (red dotted line) dramatically improved the therapeutic response to IVIG and was accompanied by a rapid increase in ΔIgG (increase in serum IgG levels 2 weeks after the start of IVIG treatment relative to pre-treatment levels). Purple square, Medical Research Council (MRC) sum score; light blue triangle, eGFR; magenta circle, anti-SRP antibody titer; green triangle, serum CK concentration; blue circle, %VC; orange diamond, ΔIgG; IVMP, intravenous methylprednisolone pulse therapy; IVIG, intravenous immunoglobulin therapy.

A 64-year-old woman with IgA nephropathy characterized by low disease activity and moderate chronic kidney disease (CKD, stage G3b; estimated glomerular filtration rate (eGFR), approximately 40 ml/min/1.73 m^2^) and asymptomatic chronic tonsillitis for over 30 years was admitted to our hospital because of progressive weakness in the extremities and hyperCKemia for 4 months. She had moderate limb and trunk muscle weakness and atrophy, which corresponded to grade 2–4 on the Medical Research Council (MRC) scale with a sum score of 50 points. Spirometry indicated mild restrictive ventilatory impairment (% predicted vital capacity (%VC), 75.5%) without symptomatic dyspnea. Laboratory results showed elevated serum CK levels (6,774 U/L) without worsening CKD. RNA immunoprecipitation (2) revealed seropositivity for anti-SRP antibodies (**Figure 1A**). MRI detected limb muscle atrophy, especially in the triceps, quadriceps, hamstrings, and adductor magnus (**Figure 1B**). Electromyography showed myopathic changes. Muscle biopsy from the left biceps demonstrated myopathic changes with active myofiber necrosis and regeneration of muscle fibers without obvious lymphocyte infiltration (**Figure 1C**), mild expression of human leukocyte antigen–ABC in scattered non-regenerating fibers (**Figure 1D**), and deposition of C5b-9 membrane attack complex in the sarcolemma (**Figure 1E**). These findings were compatible with IMNM. She was treated with three cycles of methylprednisolone pulse therapy (IVMP, 1 g/day for 3 consecutive days) followed by 1 mg/kg/day of oral prednisolone (PSL, 40 mg/day) and 50 mg/day of azathioprine. However, muscle weakness and atrophy still progressed. We added intravenous immunoglobulin therapy (IVIG, 400 mg/kg/day for 5 consecutive days). The first course of IVIG quickly normalized serum CK levels and partially improved %VC (82.8%), but limb muscle weakness and atrophy remained severe, with an MRC sum score of 46 points (**Figure 3**). We thought that her respiratory impairment was mild enough to respond to a single course of IVIG in combination with PSL and azathioprine, but her limb and trunk muscle weakness remained too severe to respond to these drugs. Therefore, we repeated IVIG every 4 weeks in combination with 40 mg/day of PSL and 50 mg/day of azathioprine. We measured ΔIgG, the change in serum IgG levels 2 weeks after the start of IVIG treatment relative to pre-treatment levels, as an indicator of IgG consumption by inflammation; ΔIgG has been used as an effective prognostic biomarker of Guillain-Barré syndrome treated with IVIG (6). Multiple rounds of IVIG gradually increased %VC to 93.6%, but severe limb muscle weakness and atrophy persisted, with an MRC sum score of 48 points, and ΔIgG remained low (**Figure 3**), indicating persistent inflammation. We suspected that chronic tonsillitis was the source of persistent inflammation, even though the patient’s chronic tonsillitis did not worsen during treatment. Finally, she underwent tonsillectomy as potential therapy for abnormal immune reaction associated with chronic tonsillitis. As shown in **Figure 2**, immunohistochemical analyses of resected tonsil sections showed chronic tonsillitis associated with macrophage activation based on hematoxylin and eosin (H&E) staining and CD68 (a marker of activated macrophages). Immunostaining for CD4 and CD8 showed T cell accumulation on the opposite side of blind crypts. Immunostaining for CD10 (a marker of germinal center B cells), CD21 (a marker of germinal center dendritic cells), CD20 (a marker of follicular B cells), and IgD (a marker of mantle zone B cells) revealed that most lymphoid follicles consisted of primary follicles with poorly developed germinal centers, rare secondary follicle formation, and an expanded mantle zone. These observations were in accordance with characteristic pathological features of chronic tonsillitis associated with IgA nephritis but not chronic tonsillitis that is not associated with IgA nephritis. (7–10) Serum anti-SRP antibody titers became undetectable after tonsillectomy (**Figure 3**) although RNA immunoprecipitation (2) did not detect anti-SRP antibodies in the resected tonsils (**Figure 1A**). Muscle weakness dramatically responded to IVIG, which was accompanied by a rapid increase in ΔIgG (**Figure 3**) and recovery of muscle volume (**Figure 1F**) and strength (grade 4–5/5 on the MRC scale with a sum score of 56 points) (**Figure 3**). The patient became able to walk without assistance. In contrast, tonsillectomy did not improve IgA nephropathy–induced CKD status (**Figure 3**). PSL was tapered to 5 mg/day and the patient was discharged. She received bimonthly IVIG (1 g/kg) to maintain muscle strength.

## Discussion

This is the first report of anti-SRP myopathy alleviated by tonsillectomy. Chronic infection has long been considered a risk factor for various disorders including autoimmune diseases, since the era of ancient Mesopotamia and Greece. (11, 12) Chronic tonsillitis has been attracting attention as a trigger of many autoimmune diseases such as IgA nephritis, IgA vasculitis, palmoplantar pustulosis, psoriasis, rheumatoid arthritis, Behçet’s disease, and myositis. Tonsillectomy has been used to alleviate these diseases. (3, 12).

In our patient, it is unlikely that chronic tonsillitis actively affected the disease status of IgA nephropathy in light of her low disease activity and kidney function already having reached a plateau at moderate CKD. In fact, tonsillectomy did not improve her IgA nephropathy–induced CKD. However, tonsillectomy did enhance the therapeutic response to IVIG for anti-SRP myopathy. We did not find evidence of anti-SRP antibody production in the patient’s tonsils, but an abnormal immune response due to chronic tonsillitis was likely because tonsillectomy was crucial to lowering serum anti-SRP antibody titers and improving the therapeutic response.

We propose two possible mechanisms to explain the improvement in therapeutic response with tonsillectomy. One possibility is that chronic tonsillitis *per se* enhances local clearance and consumption of IgG. ΔIgG varies depending on clearance and consumption of IgG by inflammation. Our patient had sustained low ΔIgG during monthly IVIG therapy, indicating the presence of persistent inflammation. Pathologic examination showed evidence of abnormal chronic inflammation in the tonsils. Another possibility is that chronic tonsillitis forms a vicious spiral of systemic abnormal autoimmune reactions. The following mechanism has been proposed to explain the abnormal immune reaction induced by chronic tonsillitis. (12) First, indigenous unmethylated bacterial nucleotides hyperactivate immune cells (B cells, T cells, and dendritic cells) that reside in the tonsils through Toll-like receptor 9. Interferon-γ and tumor necrosis factor-α produced by activated T cells reactivate these cells and expand the population of autoreactive T cells and autoantibodies-producing B cells. Finally, activated autoreactive T cells and autoantibodies damage the target tissue. According to this hypothesis, chronic tonsillitis may fuel anti-SRP antibody production in remote locations where autoreactive lymphocytes proliferate. Conversely, tonsillectomy may halt the vicious spiral of abnormal autoimmune reactions. Our findings suggest that the removal of chronically infected tissues such as the tonsils may provide a potential therapeutic effect in anti-SRP myopathy by reducing disease activity or enhancing therapeutic response.

## Data Availability Statement

The original contributions presented in the study are included in the article/supplementary material. Further inquiries can be directed to the corresponding authors.

## Ethics Statement

Investigations and interventions performed for this patient were all part of routine clinical care. Written informed consent was obtained from the patient for the publication of this case report in accordance with the Declaration of Helsinki.

## Author Contributions

TI, HT, KT, HN, MK, AK, MT, YH, SK, HD, and FT examined and treated the patient. TH and IN performed histological analysis. SS performed RNA immunoprecipitation to assess for the presence of anti-SRP antibodies. TI, HT, and FT wrote the manuscript. All authors contributed to the article and approved the submitted version.

## Funding

This study was supported partly by grants-in-aid for Scientific Research from the Ministry of Education, Culture, Sports, Science and Technology of Japan; grants from the Ministry of Health, Labour and Welfare of Japan; a grant for Strategic Research Promotion from Yokohama City University; and an Intramural Research Grant (29-4) for Neurological and Psychiatric Disorders from the National Center of Neurology and Psychiatry.

## Conflict of Interest

The authors declare that the research was conducted in the absence of any commercial or financial relationships that could be construed as a potential conflict of interest.

The remaining authors declare that the research was conducted in the absence of any commercial or financial relationships that could be construed as a potential conflict of interest.
